# A counselling line for problem and pathological gambling in South Africa: Preliminary data analysis

**DOI:** 10.1556/JBA.3.2014.017

**Published:** 2014-08-26

**Authors:** HEIDI SINCLAIR, ADELE PRETORIUS, DAN J. STEIN

**Affiliations:** ^1^Department of Psychiatry and Mental Health, University of Cape Town, Cape Town, South Africa; ^2^National Responsible Gambling Programme, South African Gambling Foundation, South Africa

**Keywords:** behaviour addictive/therapy, counselling, comorbidity, gambling/psychology, South Africa

## Abstract

*Objective:* Various countries and states have established telephone counselling lines for people with pathological or problem gambling. Data from such services may contribute to describing systematically the nature of gambling problems in a particular area. To date, however, few data have been published on such a telephone counselling line in a low or middle income country. *Method:* Data on calls to the telephone counselling line of the National Responsible Gambling Foundation of South Africa were captured over a 6-month period. Such data include socio-demographic variables, the primary reason for calling, the source of the referral, preferred method of gambling, impairment as a consequence of gambling, and history of treatment for psychiatric disorders, comorbid alcohol abuse and illicit drug use. *Results:* Calls were received from a broad range of people; the mean age of callers was 37 years, the majority were male (62%) and many were married (45%). Primary reasons for calling included the feeling of being unable to stop gambling without the help of a professional (41%), financial concerns (32%), legal problems (13%), pressure from family (10%), and suicidal thoughts (2%). The majority of callers contacted the counselling line after having heard about it by word of mouth (70%). The most common forms of gambling were slot machines (51%) and casino games (21%). Fourteen percent of callers reported having received help for other psychiatric disorders, 11% reported alcohol use disorders and 6% illicit drug use. *Conclusion:* These data from South Africa are consistent with prior research indicating that pathological and problem gambling are seen in a range of socio-demographic groups, and that such behaviour is associated with significant morbidity and comorbidity. More work is needed locally to inform younger gamblers, gamblers using the informal gambling sector, and unemployed gamblers of the existing telephone counselling lines.

## Introduction

Pathological gambling has been officially recognized in the psychiatric nomenclature for three decades. Diagnostic criteria for pathological gambling currently share similarities with those of drug and alcohol dependence, including features of diminished control, tolerance, withdrawal, and impairment in important activities (American Psychiatric Association, 2000). In the DSM-5, pathological gambling was reclassified and included in the category of ‘Substance-Related and Addictive Disorders’ (American Psychiatric Association, 2013). Like other disorders in this category, pathological gambling has been frequently associated with significant psychiatric co-morbidity, as well as with psychosocial sequelae such as legal problems (Grant & Potenza, 2007; Potenza, Steinberg, McClaughlin, Rounsaville & O’Malley, 2000) . Males with pathological gambling consistently report more problems with alcohol and drug use, while females consistently report more depression and anxiety disorders, than the general population (Sullivan, Abbot, McAvoy & Arroll, 1994).

Within the health sector, telephone counselling services constitute an important first point of contact for individuals seeking assistance. Telephone counselling services can be divided into two broad categories: generalist services that target the community as a whole and deal with a range of issues (e.g. Lifeline), and specialist services that either address a particular issue (e.g. Childline) or target a specific segment of the community. Specialist services are further subdivided into crisis counselling and referral services that usually provide anonymous counselling, often at a time of crisis, and continued support services that provide on-going counselling as required. Various countries have established telephone counselling lines for people with pathological or problem gambling (e.g. Australia, New Zealand, Sweden, UK and USA) as a feasible alternative to conventional face-to-face counselling (Griffiths, Scarfe & Bellringer, 1999; Potenza et al., 2000; Sullivan et al., 1994).

There is a small literature on telephone counselling services for pathological and problem gambling, which notes a number of potential advantages of such services. They may, for example, serve as a catalyst for encouraging individuals to make the next step towards conventional interventions. For example, Weinstock et al. (2011) evaluated 2900 callers to the West Virginia Problem Gamblers Help Network, and found that 76% of callers accepted referrals to in-person assessments, and 55% attended these appointments. Potenza et al. (2000) evaluated 562 callers to a gambling helpline serving southern New England and found that the majority of callers had never utilized professional or self-help gambling treatments and they therefore concluded that gambling helplines may be particularly useful for treatment-naïve individuals.

To our knowledge, the majority of work done on gambling counselling lines has emerged from developed countries. Abait and Folino (2008) conducted a study on callers to a gambling helpline in Argentina. They detected a high prevalence of affective disorders, suicide thoughts and attempts as well as financial and marital troubles. Some specific local characteristics related to the gambling preferences and their association to smoking were found. Their findings also supported the theory that the helpline might have a preventive effect at early stages of the course of pathological gambling and provide empirical bases for appropriate service planning in developing countries.

In South Africa, legalized gambling expanded rapidly during the latter part of the 20^th^ century, after a change in the Gambling Act 10 (Collins et al., 2011). A nationally representative survey in South Africa found that 57% of the adult population engage in some form of legal gambling, that 4-7% of the adult population have sub-threshold pathological gambling (or ‘problem gambling’), and that the prevalence rate for pathological gambling is around 1% (Quarterly Labour Force Survey, 2012). A National Responsible Gambling Foundation, established in 1998 in order to help prevent and reduce pathological gambling, established a counselling line.

The National Responsible Gambling Program (NRGP) telephone counselling line has been operational for the past 13 years and is located at its headquarters in Cape Town. It is available 24 hours a day, 365 days a year. The 0800 line is toll-free from landlines. Public landlines are easily accessible. The counsellors inform callers who phone from mobile phones that the NRGP prefer to carry the cost of the calls and therefore the counsellors call them back on their mobile phone numbers. This is the first-point of contact for expert advice on gambling for many, including individuals with problem gambling; family, friends, or work colleagues; gambling venue workers; and health professionals. The counselling line is widely advertised, using a range of methods, including stickers on slot machines and in gambling venues; newspaper, radio and television advertisements; and the yellow pages. The counselling line is operated by six counsellors, who have been trained in gambling problems, and who receive weekly supervision from a clinical psychologist and a psychiatrist. The NRGP’s treatments have also been broadened to offer more services to families either for them in their own right, or as an entire family unit.

When individuals call the NRGP counselling line, counsellors focus on developing a relationship and on providing psycho-education. They draw on the principles of motivational intervention. Where appropriate, callers are referred for a comprehensive face to face evaluation with a registered psychologist or social worker in their geographical area. The NRGP treatment network consists of 85 mental health professionals (clinical psychologists and social workers) in each of the major centres as well as various other towns in South Africa and Namibia (Collins et al., 2011). Where indicated, treatment practitioners are able to provide an evidence-based treatment program free of charge. Treatment practitioners meet annually to undergo training, and are also able to obtain consultation from a NRGP psychiatrist.

Our aim in this paper is to provide data from callers to the NRGP counselling line, describing socio-demographic and clinical features, as well as providing data on comorbidity and morbidity. We provide baseline data on callers to the counselling line, as well as information on who is not calling, so that appropriate interventions can be made in the future to encourage a larger and more representative volume of callers.

## Methods

As part of a routine procedure, the NRGP telephone counselling line records various data from callers. These data comprise the caller’s age, gender, marital status, employment status, primary reason for calling, source of referral, preferred method of gambling, sequelae of gambling such as involvement in illegal activities and history of treatment for psychiatric disorders. The Alcohol Use Disorders Identification Test (AUDIT) and the Drug Use Disorders Identification Test (DUDIT) are used to identify individuals with hazardous and harmful patterns of alcohol consumption or illicit drug use.

In accordance with the clinical literature on using audits to improve services, we retrospectively evaluated these calls, with a particular focus on who was and was not using the NRGP telephone counselling lines. This was done with the full support and encouragement of the National Responsible Gambling Foundation.

Data from calls during the last six months in 2013 were examined (n = 480). For the purpose of this analysis only first-time callers who were seeking treatment as a result of their own gambling were included. Exclusion criteria included callers younger than 18, already case-managed by the NRGP, hoax callers, family members, gamblers who contacted the line to have their self-exclusions lifted, callers with evidence of intellectual disability or language difficulties, and callers who refused to give information about themselves. Standard statistical methods were used to analyse categorical and dimensional variables.

### Ethics

The Human Research Ethics Committee of the University of Cape Town’s Faculty of Health Sciences formally approved the study.

## Results

Callers ranged in age from 18-72 years, with a mean age of 37 years, standard deviation 11. The majority of callers were male (62%), and many were married (45%) and in full-time employment (76%). The most common forms of gambling were slot machines (51%), and casino games (21%). Few calls (9%) came from callers who gambled at informal settings. The majority of callers contacted the helpline after having heard about this service by word of mouth (70%), from television or radio commercials (13%), or from the National Responsible Gambling Foundation website (13%).

Primary reasons for calling included the feeling of being unable to stop gambling without help from a professional (41%), financial concerns (32%), legal problems (13%), pressure from family (10%), and suicidal thoughts (2%). Although only a few patients called because of suicidal thoughts, it was notable that 33% (n = 93) had thoughts of suicide at the time of the telephone call. Callers called the line a mean of seven years after their gambling first began.

Gambling had led to illegal activities in 15% of callers. Among the illegal activities mentioned by callers, the following were noted: fraudulent loans, embezzlement and counterfeit cheques. Fourteen percent of callers reported having received help for other psychiatric disorders. Six percent of callers reported having received help for alcohol use disorders in the past, whereas 11% (7% of females and 13% of males) reported a current problem with alcohol use. Six percent of callers (2% of females and 8% of males) reported recent illicit drug use and the majority of these callers took stimulants (including khat, cocaine and methamphetamine).

**Table 1.** Demographics of sample

Demographics *N =* 480	*n*	% of sample
Median age	37	
Sex		
Female	182	38
Male	298	62
Marital status		
Married	216	45
Single	154	32
Divorced/Widowed	110	23
Employment status		
Employed	365	76
Unemployed	77	16
Retired	38	8
Primary reason for calling		
Loss of control	197	41
Financial trouble	154	32
Legal consequences	62	13
Family pressure	48	10
Suicide thoughts	10	2
Other	9	2
Preferred gambling method		
Slots	245	51
Casino table games	101	21
Horses	58	12
Internet	24	5
Informal forms of gambling	28	6
(iFifa/iChina/dice)		
Other	24	5

## Discussion

The main findings of this report are that in the low-middle income context of South Africa, 1) callers to a telephone counselling line for problem and pathological gambling represent a broad range of socio-demographic groups; 2) problem and pathological gambling behaviour is associated with significant morbidity and comorbidity; and 3) younger gamblers, gamblers using the informal gambling sector, and unemployed gamblers seem less likely to make use of the NRGP counselling line.

These findings are in many ways consistent with findings in more developed countries. For example, data from the Connecticut Council on Problem Gambling on the demographic profile and clinical features of problem and pathological gamblers who sought treatment from a helpline indicate that the telephone line was contacted by a broad range of gamblers, including both males (59%) and females (31%), and by married/cohabiting (45%) individuals as well as single/divorced (55%) individuals (Weinstock et al., 2011) . Such data reflect community level data on socio-demographic correlates of gambling.

The type of gambling most commonly recorded in callers (slot machines, casino games) to the NRGP telephone counselling lines largely reflects findings from national surveys of gambling behaviour in South Africa (South African National Gambling Board, National Statistics). Nevertheless, other gambling segments, particularly informal gambling (as is seen in shebeens), are also common in the country as a whole, and were less commonly reported by callers. Similarly, calls were weighted toward gamblers who were in their thirties, and those in full-time employment, whereas the South-African population as a whole is currently weighted towards the youth, and comprises a large number ofunemployed individuals (Quarterly Labour Force Survey, 2012).

Previous work on gambling telephone counselling lines, as well as from community and clinical samples has established the high morbidity and comorbidity associated with pathological and problem gambling (Ledgerwood, Steinberg, Wu & Potenza, 2005; Cunningham-Williams et al., 2005; Barry, Potenza, Steinberg & Wu, 2008). The high reported rate of suicidal ideation (33%) found here is consistent with previous international studies showing elevated rates of suicidal ideation and completed suicides among pathological gamblers (Ledgerwood et al., 2005; Pulford et al., 2009). The rate of suicidal ideation among callers to the NRGP helpline is significantly higher than the rate which was found among a nationally representative sample in South Africa, where estimated lifetime prevalence of suicidal ideation was 9.1% (Joe, Stein, Seedat, Herman & Williams, 2008) Similarly, rates of alcohol and substance use disorders were high in callers, consistent with previous work (Ledgerwood et al., 2005; Cunningham-Williams et al., 2005; Barry et al., 2008).

Callers noted several precipitants for seeking help. Similarly, previous work has shown that the majority of gamblers seek help during crisis situations such as financial difficulties, criminal charges/legal proceedings, loss of job, family break-up and attempted suicide as a result of gambling (Pulford et al., 2009).

It is unclear whether gambling precipitates suicidality or whether underlying mood disturbances lead to both gambling and suicidality. Examining the relationship between comorbidity of depression and presence of suicidality, as well as that between severity of gambling symptoms and extent of suicidality may help shed light on this question. Furthermore, all published data on the association of suicidality and gambling is from high income countries, and the nature of this relationship in low and middle income countries such as South Africa has not been explored. Despite these limitations, the results obtained are the first to explore the relationship between gambling and suicidality in South Africa, and they have clinical implications for understanding and assessing individuals with gambling disorders. It is important that individuals with gambling disorders be assessed for suicidality and comorbid psychiatric disorders, including depression, and treated for these conditions. Conversely, due to the strong relationship between gambling, suicidality and psychiatric disorders, individuals with psychiatric disorders should be screened for gambling problems.

The results have additional implications for treatment of individuals with problem or pathological gambling. Awareness of risk factors for suicidality, which overlap in part with risk factors for suicide in depression, is important in the counselling of these individuals, and may be a useful tool for helping to prevent suicide.

It is notable that twelve percent of the callers’ primary motivation for dialling the counselling line was as a result of concerns about illegal activities. Indeed, thirteen percent of female callers and 16% of male callers admitted to being involved in some form of illegal activity as a result of their gambling, consistent with the international data (Pulford et al., 2009).

The data do not address the question of whether a counselling line can effectively prevent or reduce problem and pathological gambling. Nevertheless, an average of a hundred callers per month is referred on for treatment by NRGP counsellors. At the same time, our data suggest that not all segments of society are aware of or able to access the help-line; in particular younger gamblers (18-23 years), gamblers using the informal gambling sector, and unemployed gamblers seem less likely to make use of the NRGP counselling line. Such findings are in line with national trends in health service utilization in the country as a whole, where barriers to treatment, particularly in those from low socioeconomic groups, persist (Bruwer et al., 2011).

There are a number of limitations of the current study that should be emphasized. First, the data were gathered during a single 6-month period; there may be fluctuations, often as a result of festive seasons or year-end bonuses received, in the nature of callers over the course of a year. Second, the majority of variables recorded are self-report in nature; a clinical interview may have produced different data. Callers may have minimized comorbid substance use problems in an effort to obtain access to gambling services.

Despite these limitations, this study provides relevant preliminary data on callers to a gambling telephone counselling line in a low-middle-income country. The counselling line is accessed by a broad range of individuals, many of whom have significant morbidity and comorbidity, and who are then appropriately referred for treatment. More work is needed locally on how best to encourage problem and pathological gamblers to seek help, including how best to inform younger gamblers, gamblers using the informal gambling sector, and unemployed gamblers, of the existing telephone counselling lines.
